# Barcoding Techniques Help Tracking the Evolutionary History of the Introduced Species *Pennaria disticha* (Hydrozoa, Cnidaria)

**DOI:** 10.1371/journal.pone.0144762

**Published:** 2015-12-11

**Authors:** Maria Pia Miglietta, Dean Odegard, Baptiste Faure, Anuschka Faucci

**Affiliations:** 1 Department of Marine Biology, Texas A&M at Galveston, Galveston, TX, 77553, United States of America; 2 University of Notre Dame, Notre Dame, IN, 46556, United States of America; 3 Laboratoire d’Ecologie Marine, Université de la Réunion, St Denis, La Réunion, France; 4 Department of Biology, University of Hawaii at Manoa, Honolulu, HI, 96822, United States of America; Queensland University of Technology, AUSTRALIA

## Abstract

The Christmas tree hydroid *Pennaria disticha* is listed as one of the most common introduced species in Hawaii. Firstly reported in Kaneohe Bay (Oahu) in 1928, it is now established throughout the entire archipelago, including the Northwestern Hawaiian Islands, a U.S. National Monument and World Heritage site. The Hawaiian population of *P*. *disticha* has also been reported as being the source of further introductions to Palmyra Atoll in the U.S. Line Islands. Using a phylogenetic hypothesis based on a 611 base pair fragment of the mitochondrial 16S barcoding gene, we demonstrate that *P*. *disticha* is a complex of cryptic species, rather than one species with cosmopolitan distribution. We also show that in Hawaii there are three species of *Pennaria*, rather than one introduced species. Two of these species share haplotypes with specimens from distant locations such as Florida and Panama and may have been introduced, possibly from the Atlantic Ocean. A third species could either represent a lineage with nearly cosmopolitan distribution, or another introduced species. Our dataset refutes the widely accepted idea that only one lineage of *P*. *disticha* is present in Hawaii. On the contrary, *P*. *disticha* in Hawaii may be the outcome of multiple independent introductions of several morphologically undistinguishable cryptic lineages. Our results uncover an unsuspected complexity within the very common hydroid *P*. *disticha*, and highlight the need for routine use of molecular tools, such as DNA barcoding, to improve the identification and recognition of non-indigenous species.

## Introduction

The recent increase in human mobility has increased the intentional and unintentional introduction of species worldwide. For example, at any given time, about 10,000 species travel the globe in the ballast water of ships [1]. Most introduced species do not survive to reproduction, yet some become established and a few become invasive. Those few invasive species, however, can have a significant deleterious effect on the local environment, native taxonomic assemblages, and the local economy. Once invasive species establish themselves in the new environment, they are hard to control or contain and nearly impossible to eradicate [2]. Early detection still represents the best strategy to confine a species’ introduction, and in the last decade molecular tools, such as DNA barcoding, have been useful in detecting non-indigenous species in all taxonomic groups from vertebrates to invertebrates [3–5].

The Hawaiian Archipelago has long been impacted by both marine and terrestrial introduced species. In a comprehensive report 490 marine species have been reported as non-indigenous [6], of these 35 are Hydrozoa (phylum Cnidaria). Hydrozoa are colonial invertebrates with a complex life cycle that encompasses sessile polyps, pelagic jellyfish, and planula larvae. The use of traditional taxonomy based on morphological traits of both polyps and medusae has often been deceiving and, in the last decade, has been challenged by molecular tools [7, 8]. For example, analyses based on the hydrozoan barcoding molecule (the mitochondrial 16S gene), have shown that species assumed to be cosmopolitan are often groups of cryptic species sometimes separated by several million years of evolution [9]. Conversely, specimens of the same species may look dramatically different due to morphological plasticity [9, 10]. Altogether, hydrozoan taxonomy has highly benefitted from molecular analyses, especially because the commonly used DNA barcoding molecule is effective in defining species boundaries [e.g., 11, 12, 13], and sequencing costs are increasingly affordable.

Non-indigenous Hydrozoa have been abundantly reported, but their effects on indigenous communities are often not fully understood and hardly quantified because benthic polyps and planktonic medusae are ecologically different. Thus, to fully appreciate one species’ impact, it is necessary to quantify the cumulative impact of both ontogenetic stages on benthos and plankton [14, 15]. In general, the benthic polyps compete with local species for space, while polyps and medusae prey upon larvae of invertebrates and fish [16–18]. Examples of non-indigenous Hydrozoa include the freshwater *Cordilophora caspia*, invasive in the Great Lakes region, *Blackfordia virginica*, *Moerisia* sp. and *Maeotias marginata*, reported as invasive in the San Francisco estuary [19, 20], *Maeotias marginata*, invasive in the Baltic Sea [21], *Turritopsis dohrnii*, introduced to several localities across the globe from Japan to Florida and Panama [10], and several species reported as invasive in the North Pacific and Alaska [22]. Of these introduced hydrozoan species, only *Cordilophora caspia* and *Turritopsis dohrnii* have been confirmed as such using molecular tools [10, 23, 24]. However, the way we define and recognize species boundaries in Hydrozoa arguably impacts our capability to correctly identify non-indigenous species.

*Pennaria disticha* Goldfuss, 1820, also known as the Christmas tree hydroid, is a common Hydrozoa (Cnidaria) belonging to the suborder Capitata. It forms large pinnate colonies with dark perisarcs, pink-whitish polyps, a ring of filiform tentacles at the base of the hydranth (polyp body), and a ring of capitate tentacles at the base of the hypostome (mouth). Its pinnate colonies may cause dermatitis, if contact with skin occurs [25]. It lives in shallow waters on hard substrata, and reproduces via short-lived eumedusoids (non feeding medusae). *Pennaria disticha* has a wide geographic distribution and has been reported in most warm waters around the world, including tropical and subtropical parts of the Atlantic, Indian and Pacific Oceans [26].

*Pennaria disticha* is the only species of the genus *Pennaria* reported in Hawaii [27], and is one of the most conspicuous shallow water hydroids within the archipelago [6, 27]. It has been considered introduced since its first record in 1928, when a single large colony was observed in Kaneohe Bay (Oahu) [6]. Edmondson [28] reported it as *Pennaria tiariella* and speculated that the presence of such a cosmopolitan Hydrozoa in Hawaii was due to human introduction, mostly based on the fact that it was collected from artificial substrata [28]. It was later argued that its presence in Hawaii had possibly gone unnoticed for several years and thus predated 1928 [6]. *Pennaria disticha* has since been classified as one of the most common introduced species throughout the State of Hawaii [29] and is commonly listed as introduced in Hawaii in scientific reports, Hawaiian species inventories, invertebrate guides, and popular websites. It has also been reported as established on nine reefs and atolls in the Northwestern Hawaiian Islands (NWHI), which are part of the Papahanaumokuakea Marine National Monument, a World Heritage site [30, 31], therefore considered one of few non-indigenous species with established populations throughout the entire NWHI. Moreover, the Hawaiian population of *P*. *disticha* has been reported as the source of further introductions to Palmyra Atoll in the U.S. Line Islands [32]. The National Park Service has catalogued *P*. *disticha* as an invasive species, and most agencies consider it an introduced species with potential impact on the local community. The geographic origin of this introduction has remained unknown [6, 31], but it was thought that the species arrived as part of ships’ biofouling community because the first records were from Kaneohe Bay and Pearl Harbor, two localities characterized by high maritime traffic.

In this paper we use a phylogenetic approach based on a 611 base pair (bp) fragment of the mitochondrial 16S barcoding gene to investigate the natural range of the Hydrozoa *P*. *disticha* and track the origin of its introduction to the Hawaiian Archipelago.

## Materials and Methods

### Sample collection

**Fig 1:**
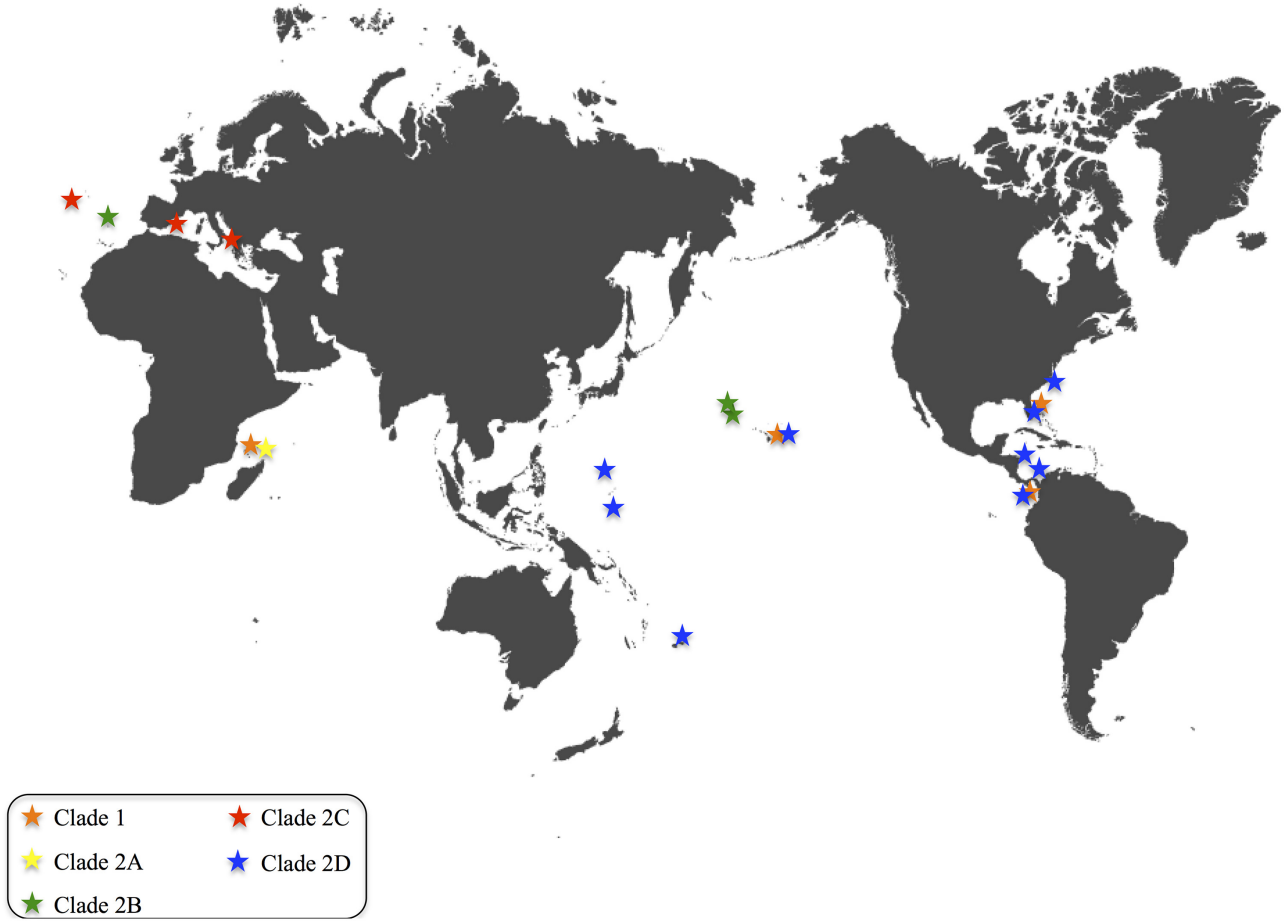
World map showing the distribution of the five *Pennaria disticha* clades, color coded as in Fig 2.

Individual colonies of *Pennaria disticha* were obtained from Pacific Islands (Hawaii, American Samoa, Guam, Chuuk), Pacific and Atlantic Panama, the Caribbean (Honduras) and Mediterranean Sea (Spain and Italy), as well as the Atlantic (Florida, North Carolina, Madeira, Azores) and Indian Oceans (Mayotte) (Fig 1). A complete list of the 81 samples, localities, voucher specimens and GenBank accession numbers (KT984672 to KT984749) is provided in Table 1.

**Table 1:** Specimens of *Pennaria disticha* herein investigated, with sequence labels, collection sites, number of specimens included, collector and year of collection, and GenBank accession numbers for 16S. (Collectors or Museum collections: AF-Anuschka Faucci, BN-Brian Nedved, BPBM-Bernice Pauahi Bishop Museum, CR-Christina Runyon, MP-Maria Pia Miglietta, NGB-Nicole Gravier-Bonnet, PS-Peter Schuchert, SP-Stefano Piraino).

Sequence labels	Collection locality	n	collection	GenBank accession no.
1, 2, 7, 10, 17, 20	Pacific, Hawaii, Oahu, Pearl Harbor	6	2011, BN	KT984718, KT984744, KT984673, KT984729, KT984740, KT984743
54	Pacific, Hawaii, Oahu, Pearl Harbor	1	1996, BPBM	KT984706
8, 9	Pacific, Hawaii, Oahu, Keehi Marina	2	2012, BN	KT984684, KT984685
3, 5, 6, 13–16	Pacific, Hawaii, Oahu, Kaneohe Bay	7	2011, CR	KT984732, KT984731, KT984734, KT984737, KT984738, KT984739, KT984730
4	Pacific, Hawaii, Oahu, Haleiwa	1	2011, AF	KT984733
12	Pacific, Hawaii, Oahu, Kewalo Basin	1	2011, AF	KT984736
11	Pacific, Hawaii, Maui	1	2011, BN	KT984735
56, 59	Pacific, Hawaii, Lisianski	2	2002, BPBM	KT984710, KT984709
55	Pacific, Hawaii, Pearl and Hermes	1	2002, BPBM	KT984707
61	Pacific, Chuuk	1	2003, AF	KT984713
88	Pacific, Guam	1	2003, NGB	KT984745
19	Pacific, American Samoa	1	2002, BPBM	KT984719
44	Pacific, Panama	1	2007, MP	KT984702
42, 91	Pacific, Panama, Las Perlas	2	2007, MP	KT984700, KT984741
72	Caribbean, Honduras	1	2004, PS	KT984717
63, 65, 66	Atlantic, Panama, Bocas del Toro	3	2007, MP	KT984746, KT984748, KT984749
23–41, 43, 45–52, 64	Atlantic, Florida, Fort Pierce	29	2011, MP	KT984674, KT984675, KT984686, KT984687, KT984688, KT984689, KT984683, KT984690, KT984691, KT984682, KT984692, KT984693, KT984694, KT984695, KT984681, KT984696, KT984697, KT984698, KT984699, KT984701, KT984680, KT984679, KT984678, KT984677, KT984703. KT984676, KT984704, KT984705, KT984747
87	Atlantic, North Carolina, Beaufort	1	2000, PS	KT984742
67	Atlantic, Portugal, Azores	1	?, PS	KT984714
57	Atlantic, Portugal, Madeira	1	2009, PS	KT984708
74–84	Mediterranean, Spain, Mallorca	11	1997, PS	KT984721, KT984720, KT984722, KT984723, KT984724, KT984725, KT984726, KT984727, KT984728, AY512533, AM088481
68, 69	Mediterranean, Italy, Taranto	2	?, SP	KT984715, KT984716
21, 58, 60	Indian, Mayotte	3	2009, NGB	KT984672, KT984712, KT984711

### Ethics statement

*Pennaria disticha* is not an endangered or protected species. Specimens from Hawaii, American Samoa, Guam, Taranto/Italy and Fort Pierce/FL were collected without the need of a permit because sampling was never conducted in a restricted marine area. Permits to collect *P*. *disticha* in Panama were granted through the Smithsonian Tropical Research Institute by the Ministry of Environment (Formerly ANAM) and the Panama Aquatic Resources Authority (ARAP), in Chuuk through the Department of Marine Resources and in Mayotte (La Reunion) through the DAF Mayotte (Direction de l'Agriculture et de la Forêt de Mayotte). DNA sequences of *P*. *disticha* from Honduras, Spain (Mediterranean Sea) and the Atlantic (North Carolina, Madeira, Azores) were obtained from extracted DNA directly provided by the Natural History Museum of Geneva, Switzerland.

### DNA sequencing

A 611bp fragment of the mitochondrial 16S gene was amplified using primers SHA 5′-ACGGAATGAACTCAAATCATGT-3′ and SHB 5′-TCGACTGTTTACCAAAAACATA-3′ [33] under the following PCR conditions: 1 min at 94°C, then 35 cycles of 94°C for 15 s, 50°C for 1:30 min and 72°C for 2:30 min with a final extension at 72°C for 5 min.

The PCR product was run on a 1.5% agarose gel stained with ethidium bromide to assay the quantity and quality (i.e., accessory bands) of the product, purified using a mixture of exonuclease I and shrimp alkaline phosphatase (ExoSAP; USB) and used as a template for double stranded sequencing at the genomic core facility at the University of Notre Dame or at the Center for Genomics, Proteomics, and Bioinformatics (University of Hawaii).

### Phylogenetic analysis

The sequences were first assembled and edited using the software Geneious 6.1.6 (Biomatters). They were then aligned using MUSCLE as implemented in Geneious 6.1.6 and then confirmed and edited by eye in MacClade 4.06. Phylogenetic analysis of the aligned sequences was performed using the maximum likelihood (ML) optimality criterion in GARLI (GUI version 0.95) and RaxML, and Bayesian inferences in MrBayes [34], as implemented in TOPALi v2 [35]. TOPALi was also used to calculate the optimal model of sequence evolution. The best-fit model suggested by TOPALi under the Bayesian Information Criterion (BIC) was TIM+G for RaxML and HKY+G for MrBayes. The ML analyses in GARLI were performed using random starting trees and default termination conditions. RaxML was used for the ML analysis using the models given above. Clade stability was assessed by ML bootstrap analyses [36] in RaxML (100 bootstrap replicates) and GARLI (100 replicates). Bayesian inference was conducted via MrBayes using the models listed above, two runs for 1,000,000 generations, and with trees being sampled every 1000 generations (the first 25% of the trees were discarded as ‘‘burnin”). Because the genus *Hydrocoryne* has been shown to be the sister taxon to *Pennaria* in a recent multigene phylogeny [37], *Hydrocoryne murensis* (genbank accession #GQ395326) was used as outgroup. Analyses were also repeated using two outgroups (*H*. *murensis* and *Cladocoryne floccosa* genbank accession # EU876554) and yielded the same topology (not shown). Phylogenetic trees were visualized and annotated with FigTree 1.4.2 [38].

**Fig 2:**
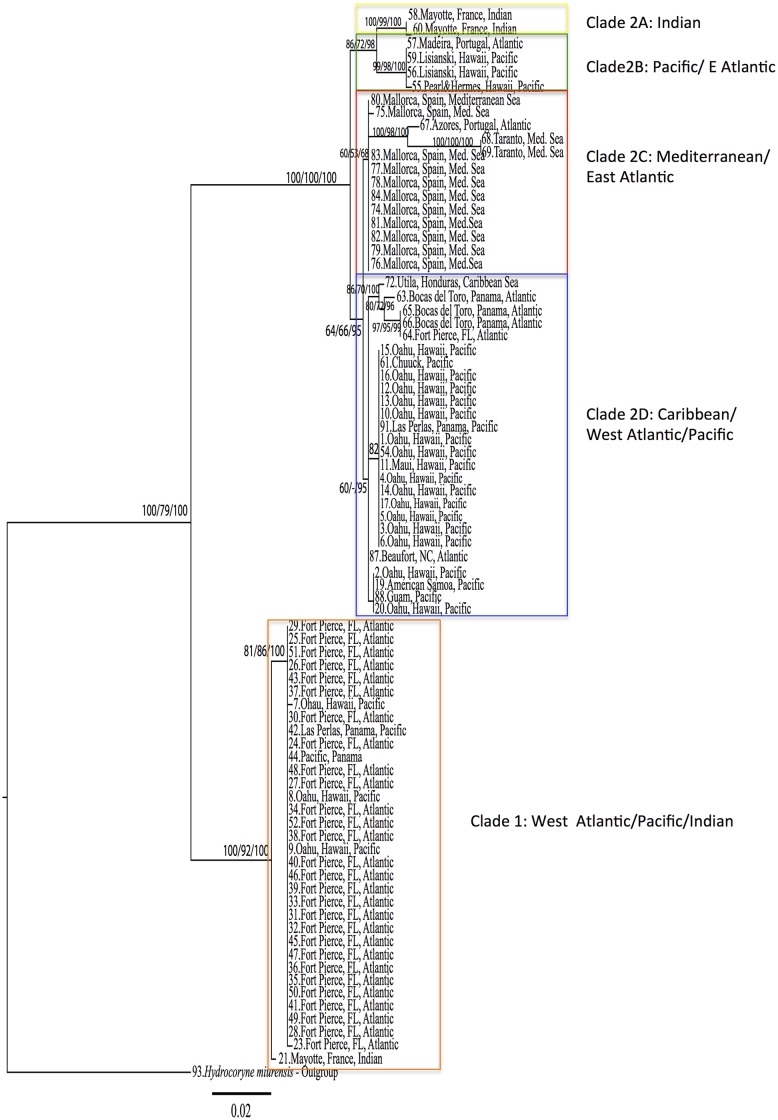
Maximum Likelihood phylogenetic hypothesis based on a 611bp fragment of the mitochondria gene 16S. Garli and RaxML runs produced the same topology. Five clades (1, 2A, 2B, 2C, 2D) are indicated by color-coded blocks. Bootstrap values and posterior probabilities are indicated at each node in the following order: Garli/RaxML/MrBayes (bootstrap values and posterior probabilities <50 not shown). See text for details on analyses.

Within- and between-group Kimura 2-parameter average distances [39] were calculated in MEGA 6.0.6 for Mac [40]. Groups were defined as the five reciprocally monophyletic clades resulting from the phylogenetic analyses (Fig 2, Results).

## Results and Discussion

The dataset is composed of 81 sequences, 80 of which belong to the ingroup (*Pennaria disticha*) and one to the outgroup (*Hydrocoryne miurensis*). All sequences but three (*H*. *murensis* GQ395326.1, *P*. *disticha* AY512533 and AM088481 (#83 and #84 in the tree, respectively)) are new and published for the first time in this paper. The data matrix had 625 characters, with missing data identified by '?' and Gaps identified by '-'. Of the 625 total characters, 479 characters were constant, 67 variable characters were parsimony-uninformative, and 79 variable characters were parsimony-informative.

The ML tree searched in GARLI and RaxML recovered the same topology (shown in Fig 2). The ingroup resulted monophyletic with bootstrap values of 100/79 (Galri/RaxML) and Bayesian posterior probability of 100 (calculated using *H*. *murensis* and *C*. *floccosa* as outgroups). The phylogenetic tree topology shows several distinct lineages within *P*. *disticha*. We identify five reciprocally monophyletic clades with levels of differentiation consistent with interspecific differentiation observed in other hydrozoan taxa [9, 10]. For simplicity of discussion, the two main clades in Fig 2 are indicated as 1 and 2. Additionally, four reciprocally monophyletic clades are also identifiable within clade 2 (2A, 2B, 2C, 2D, Fig 2). The criterion of monophyly adopted here has worked well in defining species in other hydrozoan groups [9, 10]. Notably, clades 1, 2A and 2C have high ML bootstrap support and Bayesian posterior probabilities, while clades 2C and 2D have substantially lower values (see Fig 2).

Our analysis based on the mitochondrial 16S barcoding gene shows that *Pennaria disticha* is most likely a complex of cryptic lineages separated by as much as 48bp (Table 2). More specifically, the tree topology reveals the presence of five reciprocally monophyletic clades (Fig 2), four of which exhibit broad geographic distribution. Three clades have very high bootstrap support (BS) and Bayesian posterior probabilities (PP) (clade 1, 2A and 2B). Two (clade 2C and 2D) have low BS ad PP, however they cluster specimens from non-overlapping localities and are thus discussed separately.

**Table 2:** Genetic divergence within and between clades in Fig 2 (% genetic distance (%; Kimura-2-parameter) below and number of base pairs (bp) above diagonal).

Clades	1	2	2A	2B	2C	2D
**within %**	0.001	0.018	0.002	0.001	0.010	0.004
**within bp**	0.351	9.426	1	0.5	5.209	2.354
**1**		45.116	47.647	43.934	45.092	44.727
**2**	0.088					
**2A**	0.088			10.250	14.750	14.308
**2B**	0.085		0.019		14.607	13.769
**2C**	0.088		0.088	0.085		6.516
**2D**	0.084		0.026	0.026	0.013	

The genetic distance between these five lineages ranges from 1.3% (6bp) to 8.8% (48bp) (Table 2). Based on the hydrozoan barcoding molecule (~600bp of the mitochondrial 16S gene) the levels of differentiation observed (Table 2) are consistent with interspecific differentiations observed within other hydrozoan species. For example, species belonging to *Podocoryna* are separated by 1.5% genetic distance, while species of *Hydractinia*, such as *H*. *symbiolongicarpus* and *H*. sp. from the Gulf of Mexico, are separated by as little as 1.1% [9].

We first describe each clade composition, followed by an analysis of their geographic distribution. Clade 1 (Fig 2) is represented by specimens from several locations in the Hawaiian Archipelago (Pacific), from Fort Pierce, FL (West Atlantic), one specimen from Las Perlas Island, Panama (Pacific), and one specimen from Mayotte (Indian Ocean) (Fig 2, bootstrap values 100/92 and posterior probability 100). Despite the geographic heterogeneity, this clade displays relatively low intraspecific diversity (0.1%), mostly accounted for by the single sample from the Indian Ocean (the intraspecific diversity of this clade is reduced to 0.021% when that sequence is pruned). It is worth noticing that all sequences from the Atlantic and Pacific Oceans within clade 1 share the same haplotype, while the single specimen from Mayotte is 0.7% divergent from the rest of clade 1. Although this genetic distance is small enough to fall within the level of intraspecific diversity, the possibility that the specimen from Mayotte represents a separate lineage needs to be tested through a bigger sample size.

Clade 2 is more heterogeneous and structured. Clades 2A and 2B are two sister groups with a genetic distance of 2.88% (15bp) (Table 2). Clade 2A is represented by two specimens from Mayotte (Indian Ocean), while 2B is represented by specimens from Hawaii (n = 3; Pacific) and Portugal (n = 1; East Atlantic). Notably, despite the great geographic separation between these localities, the Hawaiian and Portuguese specimens share the same haplotype.

Clade 2C is composed of specimens from the Mediterranean Sea (n = 13) and the Atlantic Azores (n = 1). Within this clade sequences from Taranto (Italy) and the Azores (Portugal) show particularly long branches (11bp between Taranto and the Azores, and 10bp between the Azores and Mallorca), which may suggest further cryptic speciation within this clade. Clade 2C, however, shows very low bootstrap values and posterior probability (60/53/68).

Clade 2D shows the highest intragroup diversity (0.4%), and, like clade 1, is represented by specimens from the West Atlantic and Pacific Ocean. This clade also has very low bootstrap support (64/66) and low posterior probability (95). Specimens from the Caribbean Sea and the West Atlantic within this clade—with the exception of one specimen from North Carolina—form a clade with bootstrap support of 86% (GARLI), 70% (RaxML) and Bayesian PP of 100%. The remaining sequences are from the Pacific (Oahu and Maui (Hawaii), Chuuk, Las Perlas (Panama), American Samoa, and Guam) and are scattered within two sub clades. The genetic distance between clade 2C and 2D is 1.3%, the lowest between the observed *Pennaria disticha* clades (Table 2).

### Geographic distribution of the lineages within the *Pennaria disticha* cryptic species complex

The pattern observed in *Pennaria disticha* resembles the one of the invasive brackish hydrozoan *Cordylophora caspia* [24]. Like *P*. *disticha*, *Cordylophora* shows multiple evolutionarily divergent lineages, each with broad geographic distribution [24]. At the same time, identical mitochondrial haplotypes are observed in disparate geographic regions (see Fig 1 for a graphic representation of each clade’s geographic distribution in *P*. *disticha*). This pattern is consistent with the hypothesis that *P*. *disticha* is composed of several (possibly five, Fig 2) cryptic species. As in *Cordilophora*, multiple lineages of *Pennaria* co-occur in several locations. Our phylogenetic hypothesis based on the hydrozoan barcoding molecule 16S, is consistent with *P*. *disticha* being an introduced species in Hawaii. It also shows that the presence of *P*. *disticha* in Hawaii may be the outcome of not one but multiple independent introductions. To clarify this point, we will discuss the presence of lineages of *P*. *disticha* in each of the sample Oceans, with special emphasis on *P*. *disticha* in Hawaii (also see Fig 1). We acknowledge the fact that these results may change once more specimens and more locations are added to our dataset.

#### Indian Ocean

Three specimen colonies were obtained from the island of Mayotte. Our analyses show that at least two very divergent lineages are present on Mayotte (clade 1B and 2A). The genetic distance between these two clades is 8.8%.

#### Mediterranean Sea

Our analyses include specimens from Mallorca (Spain) and Taranto (Italy). All of them (n = 13) fall within clade C. We conclude that only one genealogical lineage is present in the Mediterranean Sea. Besides our Mediterranean samples, clade C also includes one specimen from the Azores (Portugal, Atlantic). Notably the sequence from the Azores and those from Taranto (Italy) are significantly divergent. The resulting intragroup genetic diversity for clade C is 1% (Table 2).

#### Atlantic Ocean

Our phylogenetic hypothesis is consistent with the presence of four lineages of *P*. *disticha* in the Atlantic Ocean. One specimen from Madeira and one specimen from the Azores are the only samples from the East Atlantic and their sequences fall within two separate lineages (clades 2B and 2C, genetic distance 2.8%, Fig 2). In the West Atlantic there are very distinct lineages (clades 1 and 2D, genetic distance 8.4%, Fig 2). Clade 1 includes specimens from Fort Pierce, Florida (n = 28), while clade 2D includes specimens from Bocas del Toro, Panama (n = 3), Fort Pierce, Florida (n = 1), Beaufort, North Carolina (n = 1), and Honduras (n = 1).

#### Pacific Ocean

In the Pacific there are three lineages (clades 1, 2B and 2D, Fig 2). The majority of specimens from Oahu, Hawaii (n = 15), and the specimen from American Samoa, together with specimens from Guam, Maui, and Chuuk, and one specimen from Las Perlas (Panama), fall within clade 2D.

The three remaining specimens from Oahu, Hawaii together with one from Las Perlas, Panama fall within clade 1. All specimens from Lisianski, NWHI (n = 2) and Pearl & Hermes, NWHI (n = 1) fall within clade 2B.

### Hawaiian Archipelago and putative introduced species

The Hawaiian Archipelago is home to three lineages within the *P*. *disticha* species complex. This is clearly in contradiction with the widely accepted idea that only one introduced species is present throughout the archipelago. Two species (clade 1 and 2B, Fig 2) share haplotypes with specimens from distant locations in the Atlantic Ocean such as Fort Pierce (FL), and Madeira (Portugal), leading us to think that they may be the result of human-mediated introductions [10]. Also, Hawaiian specimens in clade 1 share haplotypes with specimens from Las Perlas, an island offshore of Panama City in the Pacific Ocean. Panama City is a busy port with dense maritime traffic (and thus high potential for species introductions) and direct shipping routes to Hawaii (for an estimate of boat traffic between Panama and Hawaii, see Concepcion et al. [41]). The third lineage within the *P*. *disticha* complex (clade 2D) is also found in other Pacific localities such as Panama, Guam, Chuuk, and American Samoa, as well as in the Caribbean and the Atlantic. Most of the specimens collected on Oahu, Hawaii, fall within this heterogeneous clade lending support to the hypothesis that this is the most abundant species of *Pennaria* found on Oahu. The fact that all Atlantic specimens from Fort Pierce (FL), Bocas del Toro (Panama) and Hutila (Honduras) within clade 2D form a well-supported clade separated by ~0.9% genetic distance from the other Pacific samples, may indicate a finer structure that needs further analyses and deeper sampling to disentangle.

## Conclusions

Our dataset shows that *Pennaria disticha* is a complex of at least five lineages, with genetic divergences ranging from 1.3% to 8.4%. Although more sampling and more loci are necessary to improve species boundary determination, *P*. *disticha* should be considered a complex of cryptic species rather than a single species with cosmopolitan distribution.

Notably, in Hawaii there are at least three cryptic species, two of which may be the outcome of independent human-mediated introductions in the archipelago, possibly originating in the Atlantic Ocean (clade 1 and clade 2B). A third lineage (clade 2D) may either have a true cosmopolitan distribution or may be the outcome of a distinct introduction event, but further evidence is needed.

*Pennaria disticha* has also been reported as introduced in South Africa [42] and on Palmyra Atoll in the Pacific [32], and as cryptogenic in the harbor of Cádiz, on the southern coast of the Iberian Peninsula, Spain [43]. In the light of our findings it is unclear which lineage is in these localities. Our dataset uncovers an unsuspected complexity that highlights the notion that morphology alone is insufficient when identifying non-native species of Hydrozoa and possibly other taxonomic groups (see for example Concepcion et al. [41] on the snowflake coral *Carijoa* in Hawaii). It also emphasizes the importance of molecular tools such as DNA barcoding in accurate species identification. Given the affordable cost of DNA barcoding, it is desirable that such tools be broadly applied to confirm and further investigate reports of introduced species.

## References

[1] Carlton JT. The scale and ecological consequences of biological invasions in the world’s oceans In: Sandlund PJS O. T., and Viken Å., editor. Invasive Species and Biodiversity Management. Dordrecht: Kluwer Academic Publishers; 1999 p. 195–212.

[2] Bax N, Williamson A, Aguero M, Gonzalez E, Geeves W. Marine invasive alien species: a threat to global biodiversity. Marine Policy. 2003;27:313–23. 10.1016/S0308-597X(03)00041-1

[3] Blanchet S. The use of molecular tools in invasion biology: an emphasis on freshwater ecosystems. Fisheries Management and Ecology. 2012;19(2):120–32. 10.1111/j.1365-2400.2011.00832.x WOS:000301429900004.

[4] Darling JA, Blum MJ. DNA-based methods for monitoring invasive species: a review and prospectus. Biological Invasions. 2007;9(7):751–65. 10.1007/s10530-006-9079-4 WOS:000249443400001.

[5] Phair N, Barendse J, Smith MKS, von der Heyden S. Molecular analyses confirm genetically distinct populations of two indigenous estuarine fish species in an isolated coastal lake: implications for the management of introduced ichthyofauna. Conservation Genetics. 2015;16(4):801–9. 10.1007/s10592-015-0701-9 WOS:000357285500004.

[6] Carlton JT, Eldredge LG. Marine Bioinvasions in Hawaii: The Introduced and Cryptogenic Marine and Estuarine Animals and Plants of the Hawaiian Archipelago. Honolulu: Bernice P. Bishop Museum, 2009.

[7] Miglietta MP, Faucci A, Santini F. Speciation in the Sea: Overview of the Symposium and Discussion of Future Directions. Integrative and Comparative Biology. 2011;51(3):449–55. WOS:000293908300012. 10.1093/icb/icr024 21593140

[8] Santini F, Miglietta MP, Faucci A. Speciation: Where Are We Now? An Introduction to a Special Issue on Speciation. Evolutionary Biology. 2012;39(2):141–7. WOS:000304659000001.

[9] Miglietta MP, Schuchert P, Cunningham CW. Reconciling genealogical and morphological species in a worldwide study of the Family Hydractiniidae (Cnidaria, Hydrozoa). Zoologica Scripta. 2009;38(4):403–30. WOS:000267130400004.

[10] Miglietta MP, Lessios HA. A silent invasion. Biological Invasions. 2009;11(4):825–34. 10.1007/S10530-008-9296-0 WOS:000263785700005.

[11] Haddad MA, Bettim AL, Miglietta MP. Podocoryna loyola, n. sp (Hydrozoa, Hydractiniidae): a probably introduced species on artificial substrate from southern Brazil. Zootaxa. 2014;3796(3):494–506. WOS:000336088100005. 10.11646/zootaxa.3796.3.5 24870689

[12] Moura CJ, Cunha MR, Porteiro FM, Rogers AD. The use of the DNA barcode gene 16S mRNA for the clarification of taxonomic problems within the family Sertulariidae (Cnidaria, Hydrozoa). Zoologica Scripta. 2011;40(5):520–37. 10.1111/J.1463-6409.2011.00489.X WOS:000294611900007.

[13] Schuchert P. High genetic diversity in the hydroid Plumularia setacea: A multitude of cryptic species or extensive population subdivision? Mol Phylogenet Evol. 2014;76:1–9. 10.1016/J.Ympev.2014.02.020 WOS:000336820800001. 24602986

[14] Boero F, Bouillon J, Gravili C, Miglietta MP, Parsons T, Piraino S. Gelatinous plankton: irregularities rule the world (sometimes). Marine Ecology Progress Series. 2008;356:299–310. 10.3354/Meps07368 WOS:000254963900026.

[15] Gili JM, Coma R. Benthic suspension feeders: their paramount role in littoral marine food webs. Trends in Ecology & Evolution. 1998;13(8):316–21. 10.1016/S0169-5347(98)01365-2 WOS:000075004300008. 21238320

[16] Costello JH, Colin SP. Prey resource use by coexistent hydromedusae from Friday Harbor, Washington. Limnol Oceanogr. 2002;47(4):934–42. WOS:000176931700002.

[17] Miglietta MP, Rossi M, Collin R. Hydromedusa blooms and upwelling events in the Bay of Panama, Tropical East Pacific. Journal of Plankton Research. 2008;30(7):783–93. 10.1093/Plankt/Fbn038 WOS:000257964700005.

[18] Rossi S, Bramanti L, Broglio E, Gili JM. Trophic impact of long-lived species indicated by population dynamics in the short-lived hydrozoan Eudendrium racemosum. Marine Ecology Progress Series. 2012;467:97–111. 10.3354/Meps09848 WOS:000310270500008.

[19] Mills CE, Rees JT. New observations and corrections concerning the trio of invasive hydromedusae Maeotias marginata, (= M-inexpectata), Blackfordia virginica, and Moerisia sp in the San Francisco Estuary. Scientia Marina. 2000;64:151–5. WOS:000167332700017.

[20] Mills CE, Sommer F. Invertebrate Introductions in Marine Habitats—2 Species of Hydromedusae (Cnidaria) Native to the Black-Sea, Maeotias-Inexspectata and Blackfordia-Virginica, Invade San-Francisco Bay. Marine Biology. 1995;122(2):279–88. WOS:A1995QX95300013.

[21] Vainola R, Oulasvirta P. The first record of Maeotias marginata (Cnidaria, Hydrozoa) from the Baltic Sea: A Pontocaspian invader. Sarsia. 2001;86(4–5):401–4. WOS:000173717900008.

[22] Ray GL. Invasive Marine and Estuarine Animals of the Pacific Northwest and Alaska. U.S. Army Engineer Research and Development Center Vicksburg, MS, Program A-ANSR; 2005 SEP 2005. Report No.: ERDC/TN ANSRP-05-6.

[23] Darling JA, Folino-Rorem NC. Genetic analysis across different spatial scales reveals multiple dispersal mechanisms for the invasive hydrozoan Cordylophora in the Great Lakes. Mol Ecol. 2009;18(23):4827–40. WOS:000271904400008. 10.1111/j.1365-294X.2009.04405.x 19889038

[24] Folino-Rorem NC, Darling JA, D'Ausilio CA. Genetic analysis reveals multiple cryptic invasive species of the hydrozoan genus Cordylophora. Biological Invasions. 2009;11(8):1869–82. WOS:000269529100011.

[25] Tezcan OD, Sarp S. An unusual marine envenomation following a rope contact: A report on nine cases of dermatitis caused by Pennaria disticha. Toxicon. 2013;61:125–8. 10.1016/J.Toxicon.2012.10.019 WOS:000314146400015. 23174519

[26] Bouillon J, Medel MD, Pages F, Gili JM, Boero F, Gravili C. Fauna of the Mediterranean Hydrozoa. Scientia Marina. 2004;68:5–449. WOS:000224982900002.

[27] Calder DR. Some anthoathecate hydroids and limnopolyps (Cnidaria, Hydrozoa) from the Hawaiian archipelago. Zootaxa. 2010;(2590):1–91. WOS:000281478800001.

[28] Edmondson CH. Reef and Shore Fauna of Hawaii. Honolulu: Bishop Museum Press; 1933 295 p.

[29] Coles SL, Kandel FLM, Reath PA, Longenecker K, Eldredge LG. Rapid assessment of nonindigenous marine species on coral reefs in the main Hawaiian Islands. Pac Sci. 2006;60(4):483–507. 10.1353/Psc.2006.0026 WOS:000240665600005.

[30] See K, Godwin S, Menza C. Nonindigenous and Invasive Species In: Desch TW A., Brainard R., Friedlander A., Christensen J., editor. A Marine Biogeographic Assessment of the Northwestern Hawaiian Islands NOAA Technical Memorandum NOS NCCOS 84 Prepared by NCCOS’s Biogeography Branch in cooperation with the Office of National Marine Sanctuaries Papahanaumokuakea Marine National Monument. Silver Spring 2009 p. 275–90.

[31] Godwin S, Rodgers KS, Jokiel PL. Reducing Potential Impact of Invasive Marine Species in the Northwestern Hawaiian Islands Marine National Monument Hawaii Institute of Marine Biology, Administration NHIMNM; 2006.

[32] Knapp IS, Godwin LS, Smith JE, Williams CJ, Bell JJ. Records of non-indigenous marine species at Palmyra Atoll in the US Line Islands. Marine Biodiversity Records. 2011;4:7 10.1017/S1755267211000078

[33] Cunningham CW, Buss LW. Molecular Evidence for Multiple Episodes of Pedomorphosis in the Family Hydractiniidae. Biochem Syst Ecol. 1993;21(1):57–69. 10.1016/0305-1978(93)90009-G WOS:A1993KN69600008.

[34] Ronquist F, Teslenko M, van der Mark P, Ayres DL, Darling A, Hohna S, et al MrBayes 3.2: Efficient Bayesian Phylogenetic Inference and Model Choice Across a Large Model Space. Systematic Biology. 2012;61(3):539–42. 10.1093/sysbio/sys029 WOS:000303336200013. 22357727 PMC3329765

[35] Milne I, Lindner D, Bayer M, Husmeier D, McGuire G, Marshall DF, et al TOPALi v2: a rich graphical interface for evolutionary analyses of multiple alignments on HPC clusters and multi-core desktops. Bioinformatics. 2009;25(1):126–7. 10.1093/bioinformatics/btn575 WOS:000261996400021. 18984599 PMC2638937

[36] Felsenstein J. Confidence-Limits on Phylogenies—an Approach Using the Bootstrap. Evolution. 1985;39(4):783–91. WOS:A1985APJ8100007. 28561359 10.1111/j.1558-5646.1985.tb00420.x

[37] Nawrocki AM, Schuchert P, Cartwright P. Phylogenetics and evolution of Capitata (Cnidaria: Hydrozoa), and the systematics of Corynidae. Zoologica Scripta. 2010;39(3):290–304. WOS:000276661200007.

[38] Rambaut A. FigTree 1.4.2 ed University of Edinburgh 2014 p. Tree Figure Drawing Tool.

[39] Kimura M. A Simple Method for Estimating Evolutionary Rates of Base Substitutions through Comparative Studies of Nucleotide-Sequences. J Mol Evol. 1980;16(2):111–20. 10.1007/Bf01731581 WOS:A1980KW57300003. 7463489

[40] Tamura K, Stecher G, Peterson D, Filipski A, Kumar S. MEGA6: Molecular Evolutionary Genetics Analysis Version 6.0. Mol Biol Evol. 2013;30(12):2725–9. 10.1093/Molbev/Mst197 WOS:000327793000019. 24132122 PMC3840312

[41] Concepcion GT, Kahng SE, Crepeau MW, Franklin EC, Coles SL, Toonen RJ. Resolving natural ranges and marine invasions in a globally distributed octocoral (genus Carijoa). Marine Ecology Progress Series. 2010;401:113–27. 10.3354/Meps08364 WOS:000276021600010.

[42] Mead A, Carlton JT, Griffiths CL, Rius M. Revealing the scale of marine bioinvasions in developing regions: a South African re-assessment. Biological Invasions. 2011;13(9):1991–2008. WOS:000296348000005.

[43] Megina C, Gonzalez-Duarte MM, Lopez-Gonzalez PJ, Piraino S. Harbours as marine habitats: hydroid assemblages on sea-walls compared with natural habitats. Marine Biology. 2013;160(2):371–81. 10.1007/S00227-012-2094-3 WOS:000313728400011.

